# An Ensemble Network for High-Accuracy and Long-Term Forecasting of Icing on Wind Turbines

**DOI:** 10.3390/s24248167

**Published:** 2024-12-21

**Authors:** Jiazhi Dai, Mario Rotea, Nasser Kehtarnavaz

**Affiliations:** 1 Department of Electrical and Computer Engineering, University of Texas at Dallas, Richardson, TX 75080, USA; kehtar@utdallas.edu; 2 Center for Wind Energy, University of Texas at Dallas, Richardson, TX 75080, USA; rotea@utdallas.edu; 3 Department of Mechanical Engineering, University of Texas at Dallas, Richardson, TX 75080, USA

**Keywords:** icing prediction on wind turbines, icing forecast using SCADA data, ensemble CNN-TCN-GRU network

## Abstract

Freezing of wind turbines causes loss of wind-generated power. Forecasting or prediction of icing on wind turbine blades based on SCADA sensor data allows taking appropriate actions before icing occurs. This paper presents a newly developed deep learning network model named PCTG (Parallel CNN-TCN GRU) for the purpose of high-accuracy and long-term prediction of icing on wind turbine blades. This model combines three networks, the CNN, TCN, and GRU, in order to incorporate both the temporal aspect of SCADA time-series data as well as the dependencies of SCADA variables. The experimentations conducted by using this model and SCADA data from three wind turbines in a wind farm have generated average prediction accuracies of about 97% for prediction horizons of up to 2 days ahead. The developed model is shown to maintain at least 95% prediction accuracy for long prediction horizons of up to 22 days ahead. Furthermore, for another wind farm SCADA dataset, it is shown that the developed PCTG model achieves over 99% icing prediction accuracy 10 days ahead.

## 1. Introduction

The share of wind energy among various energy sources has grown significantly to 7.8% of the world’s energy consumption [1]. Wind energy worldwide has now grown to more than 1000 GW, with most of the wind energy growth occurring in China and the United States [2]. According to the VTT Technical Research Center [3], approximately 20% of all wind turbine installations are in cold climate regions. In these regions, icing of wind turbines has a significant negative impact on the power generation capacity of wind farms. For example, wind turbine blade icing changes the aerodynamic shape of its surface, thus reducing its power production. It could also lead to stalling of the blades due to ice buildup. Also, because of the non-uniformity of icing, the blades would bear unbalanced loads, resulting in rotor imbalance and thus increasing the chance of structural damage. Furthermore, ice on the blades may break off and fly away at high speeds, posing a potential safety hazard to the equipment and operating personnel.

There are many works in the literature related to the detection of icing on wind turbines using SCADA data, e.g., [4,5,6,7,8]. Detection involves obtaining whether icing exists at the current time. This work addresses icing prediction, which is different than icing detection as it involves forecasting the presence of icing at a future time. Similar to the icing detection problem, SCADA data and weather data are used here as input for icing prediction. The capability to predict icing on wind turbines ahead of time would allow taking appropriate actions such as shifting electricity production to other sources of energy, shutting down the equipment to avoid damage, or turning on heaters for those turbines that are equipped with them.

The literature on modern icing prediction or forecasting on wind turbines covers the use of deep learning networks based on SCADA (Supervisory Control and Data Acquisition) data without requiring the installation of new sensors other than those used for collecting SCADA data. In the review article [9], we discussed the existing techniques for icing prediction together with their shortcomings. In [10,11], our research team introduced a prediction framework to forecast icing on wind turbines and an entire wind farm based on SCADA data via Temporal Convolutional Network (TCN) predictors. An average prediction accuracy of about 80% was reported in [10]. In this paper, we have developed a new deep neural network model for the purpose of achieving high icing prediction accuracies at long prediction horizons.

**Figure 1:**
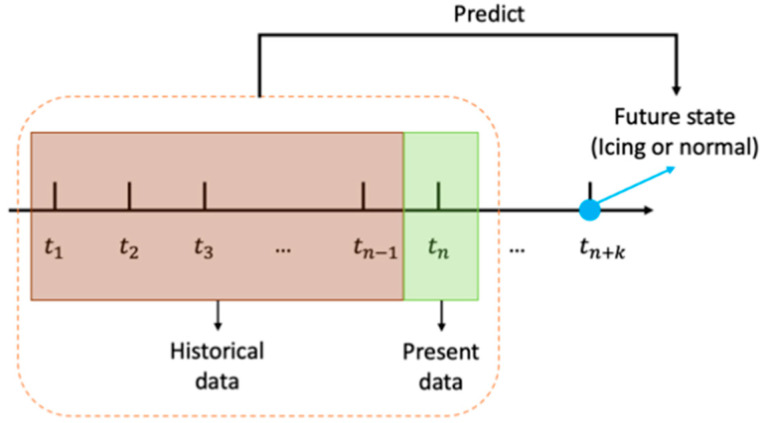
Prediction or forecasting at a future time.

In deep learning-based approaches to icing prediction, as depicted in Figure 1, the current and previous values of the variables or features in SCADA data are used as the input to a deep learning network to predict one of the two states of normal operation and icing at a future time as the network output.

Previous papers on icing prediction have utilized two major types of network models to establish the relationship between historical data and future icing states. The first type has focused on capturing the temporal aspect of SCADA data, e.g., [12,13,14]. However, these models mainly capture the temporal aspect of the SCADA features and do not take into consideration the spatial relationships between the features such as temperature, humidity, and wind speed. The second type of network models has focused on utilizing multiple learners to capture patterns between historical data and future icing states, e.g., [15,16,17,18,19]. By using multiple learners, the nonlinear relationships between the SCADA features are more effectively captured. However, such models normally assume that the input features are independent, and their temporal dependency is not taken into consideration, thus making the outcome highly dependent on the selected SCADA features. In a recent paper [20], icing prediction was performed by capturing spatial relationships between SCADA data features. It is often challenging for such models to ensure that the captured relationships between the features are meaningful, especially for the features that are influenced by the climate, such as temperature, humidity, and wind speed.

As discussed in detail in [9], among the deep learning networks that have been applied to icing prediction, in general, higher accuracies are reported with combined networks. A combined or ensemble network usually consists of the use of two networks. Different ways are considered to integrate such two networks for capturing both the temporal and spatial information in SCADA data. For example, the CNN (Convolutional Neural Network) and GRU (Gated Recurrent Unit) were used together in [21], or bidirectional LSTM (Long Short-Term Memory) and an SVM (Support Vector Machine) were used together in [19]. Here, it is important to note that certain limitations exist in the existing papers when it comes to comparing their results, including differences in SCADA features used as input, differences in prediction horizons, and the use of different datasets that are not publicly available.

The main contribution of this paper lies in developing a new deep learning architecture that combines local temporal features, their long-term relationships, and their global dependency. Compared with the existing models used for icing prediction, this new model offers higher icing prediction accuracies over longer prediction horizons.

The remaining sections of this paper are organized as follows. Section 2 introduces the ensemble model named PCTG towards achieving high-accuracy and long-term prediction of icing on wind turbine blades. Then, Section 3 provides a description of the SCADA features used and their preprocessing. Furthermore, the experimental setup together with the evaluation metrics are stated in this section. Section 4 presents the experimental results and their discussion in terms of prediction performance and computational aspects. Finally, the conclusions and future enhancements are mentioned in Section 5.

## 2. Ensemble Network PCTG

**Figure 2:**
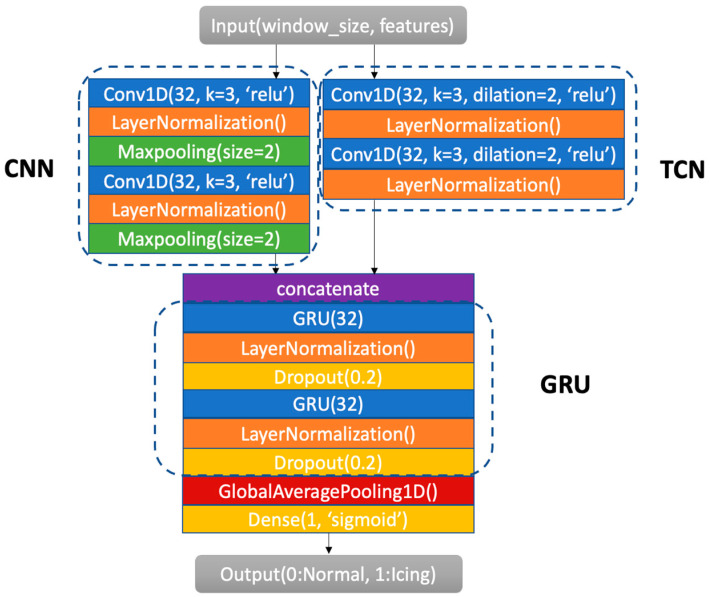
Architecture of the developed Parallel CNN-TCN GRU network named PCTG for high-accuracy prediction of icing on wind turbines.

In this paper, a new network is introduced for the prediction of icing by adding CNN and GNU layers to our previously developed TCN. Figure 2 shows the architecture of this ensemble network. The incorporation of CNN and GRU layers into the TCN allows for the more effective capture of relationships between SCADA features. The combination of these networks is named PCTG (Parallel CNN-TCN GRU) network. The PCTG uses the CNN and TCN in parallel and then integrates their outputs into the GRU. All three of these networks have been previously used for prediction tasks in different applications. The CNN has been used to capture local changes or to extract local temporal features. The TCN has been used to capture long-term dependencies through dilated convolutions. The GRU has been used to capture global time dependency via a gate mechanism. These networks have been combined in this work to take advantage of their complementary prediction attributes. Further details of this new architecture are stated next.

### 2.1. Objective Function

Wind turbine icing prediction can be viewed as a sequential two-class classification problem. For such problems, binary cross-entropy is commonly used as the loss function. In other words, the output probability that minimizes the following loss function is obtained:

(1) $$Min-\frac{1}{N}\sum_{i=1}^{N}\left[y_{i}\log\left(P\left(\hat{y_{i}}\right)\right)+\left(1-y_{i}\right)\log\left(1-P\left(\hat{y_{i}}\right)\right)\right]$$

where *N* represents the number of data samples, $y_{i}$ true icing label (0 or 1), and $P\left(\hat{y_{i}}\right)$ predicted probability by the model (a value between 0 and 1).

Consider a dataset represented by $X_{n}=\{x_{n,1},x_{n,2},\ldots ,x_{n,T}\}$, where $X_{n}$ denotes the input corresponding to the *n*th SCADA feature or variable. $o_{c,t}$ denotes the CNN, TCN, or GRU output values of channel *c* at time *t*.

(2) $$o_{c,t_{cnn}}=CNN\left({X_{1},X_{2},\ldots ,X}_{n}\right)$$

(3) $$o_{c,t_{tcn}}=TCN\left({X_{1},X_{2},\ldots ,X}_{n}\right)$$

(4) $${X^{\prime }}_{c,t_{concat}}=[o_{c,1},o_{c,2},\ldots ,o_{c,t_{cnn}},o_{c,1},o_{c,2},\ldots ,o_{c,t_{tcn}}]$$

(5) $$o_{c,t_{gru}}=GRU\left({X^{\prime }}_{1,t_{concat}},{X^{\prime }}_{2,t_{concat}},\ldots ,{X^{\prime }}_{c,t_{concat}}\right)$$

(6) $$P\left(\hat{y_{i}}\right)=Dense(o_{1,t_{gru}},o_{2,t_{gru}},\ldots ,o_{c,t_{gru}})$$

The outputs for the CNN and TCN modules process the input SCADA data independently, and then they are concatenated and fed into the GRU module to obtain predicted probabilities.

### 2.2. CNN Module

The CNN module consists of two CNN layers aimed at capturing local temporal patterns in a sequence or time series. Given a dataset $X_{n}=\{x_{n,1},x_{n,2},\ldots ,x_{n,T}\}$, we assume $K_{n}=\{k_{1,n},k_{2,n},k_{3,n}\}$, where $K_{n}$ denotes the CNN kernel function associated with the *n*th SCADA feature. Convolution is used for extracting local attributes of the features by sliding a commonly used convolution kernel of size 3 [22] over input data according to

(7) $$h_{n,t^{\prime }}=\sum_{i=0}^{2}x_{n,t+i}*k_{i+1,n}$$

Normalization and maxpooling layers are then added for the purpose of stabilizing the gradient computation and making the differences more prominent. In other words,

(8) $$h_{c,t_{cnn}}=Layer\;Normalization(\phi_{c}\left(h_{1,t^{\prime }},h_{2,t^{\prime }},\ldots ,h_{n,t^{\prime }}\right))$$

(9) $$\max h_{c,t_{\mathrm{cnn}}}=\underset{i}{\max}\left(h_{c,i},h_{c,i+1}\right),i=1,3,5,\ldots ,t-1$$

where $h_{n,t^{\prime }}$ denotes the *t*’th convolutional outcome of $X_{n}$ and $K_{n}$ for the *n*th SCADA feature, $h_{c,t_{cnn}}$ the *t*’th layer normalization of the kernel function associated with $h_{n,t^{\prime }}$ for channel *c*. Finally, ReLU (Rectified Linear Unit) is utilized for the nonlinearity mapping to the output

(10) $$o_{c,t_{cnn}}=ReLU(maxh_{c,t_{cnn}})$$

Essentially, the use of the CNN allows local patterns of features in a time series to be extracted via convolution and maxpooling operations.

### 2.3. TCN Module

The TCN module consists of two TCN layers to extract long-term temporal dependencies. Dilated convolutions are carried out as follows:

(11) $${h^{\prime }}_{n,t^{\prime }}=\sum_{i=0}^{2}x_{n,t+2i}*{k^{\prime }}_{i+1,n}$$

Layer normalization is then applied to stabilize the gradient computation, which is

(12) $${h^{\prime }}_{c,t_{tcn}}=Layer\;Normalization(\phi c\left({h^{\prime }}_{1,t^{\prime }},{h^{\prime }}_{2,t^{\prime }},\ldots ,{h^{\prime }}_{n,t^{\prime }}\right))$$

where ${h^{\prime }}_{n,t^{\prime }}$ represents the *t*’th convolutional outcome of $X_{n}$ and $K_{n}$ for the *n*th SCADA feature, ${h^{\prime }}_{c,t_{tcn}}$ the *t*’th layer normalization of the kernel function associated with $h_{n,t^{\prime }}$ for channel *c*. Finally, ReLU (Rectified Linear Unit) is utilized for the nonlinearity mapping to the output

(13) $${o^{\prime }}_{c,t_{tcn}}=ReLU({h^{\prime }}_{c,t_{tcn}})$$

By dilated convolutions, the extracted patterns by the TCN are made more global-capturing long-term dependencies in a time series.

### 2.4. GRU Module

The outputs from the CNN and TCN modules are concatenated and inputted into a GRU module. Let the input to the GRU module be

(14) $${X^{\prime }}_{c,t_{concat}}=[o_{c,1},o_{c,2},\ldots ,o_{c,t_{cnn}},{o^{\prime }}_{c,1},{o^{\prime }}_{c,2},\ldots ,{o^{\prime }}_{c,t_{tcn}}].$$

The GRU module consists of two layers. Each layer comprises two gates: an update gate and a reset gate. These two gates maintain and adjust the state of the network by controlling the flow of information towards capturing the dependencies in time-series data. The GRU computation can be expressed as

(15) $$z_{c,t}=\emptyset (W_{z}\cdot \left[h_{c,t-1},{x^{\prime }}_{c,t}\right]+b_{z})$$

(16) $$r_{c,t}=\emptyset (W_{r}\cdot \left[h_{c,t-1},{x^{\prime }}_{c,t}\right]+b_{r})$$

(17) $${\hat{h}}_{c,t}=tanh(W_{h}\cdot \left[{r_{c,t}\cdot h}_{c,t-1},{x^{\prime }}_{c,t}\right]+b_{h})$$

(18) $$h_{c,t}=\left(1-z_{c,t}\right){\cdot h}_{c,t-1}+z_{c,t}\cdot {\hat{h}}_{c,t}$$

where $z_{c,t}$ represents the update gate determining how much of the information from the previous time step is passed to the current time step towards capturing long-term dependencies;$r_{c,t}$ represents the reset gate determining how much of the hidden state information from the current input to the previous time step is discarded towards capturing short-term dependencies; ${\hat{h}}_{c,t}$ denotes the current memory generated by combining the current input and the hidden state of the previous time step adjusted via the reset gate; $h_{c,t}$ denotes the hidden state, which is generated by balancing the hidden state of the previous time step and the current memory content via the update gate; $W_{z}$, $W_{r}$, and $W_{h}$ denote weight matrices for the update gate, the reset gate, and the current memory, respectively. Layer normalization is applied to stabilize the gradient computation, and the dropout layers are applied to avoid model overfitting, which is

(19) $$o_{c,t}=Dropout(Layer\;Normalization\left(h_{c,t}\right))$$

The GRU module controls the flow of data and updates the hidden states through a mechanism of resetting and updating gates. In other words, after concatenating the CNN and TCN outputs, the GRU first captures all prominent local attributes and then gradually fits their trend within a long-term relationship. As a result, compared with the CNN-GRU, the long-term trend of a time series gets more effectively captured, and thus a more accurate prediction of a far future time can be made. Compared with the TCN, this is equivalent to setting some threshold points in advance as a regularization term to prevent overfitting. The above combined network of the CNN, TCN, and GRU forms a new ensemble network named PCTG (Parallel CNN-TCN GRU).

## 3. Data Preprocessing and Experiment Settings

For the experiments conducted, the SCADA data of three wind turbines of a wind farm used in [10] were considered. The accuracy and stability of the PCTG were compared with four previous prediction models. The same feature selection and ice labeling methods in [10] were used here. The weather data corresponding to temperature and relative humidity were also added to the SCADA data as additional features. The dataset examined contains 14,000 data samples of 12 SCADA variables/features, as reported in [10], at a sampling rate of one sample per 10 min from January 2023 to April 2023. Table 1 includes all the features or variables used as input for the ensemble network PCTG.

Based on the ice labeling rules in [10], about 35% of the data of the winter season from January to April generated icing state labels. For data balancing, the number of normal operation data samples was chosen to be the same as the number of icing data samples. The ratio of training to testing was set to 80% to 20% randomly chosen with no overlap between them. Table 2 lists the rules used in [8] for labeling data samples as ice or normal states, and Table 3 lists the number of data samples used for the training and testing.

### 3.1. Baseline Models

In this study, the following baseline models were compared to our newly developed model: 1D-CNN, LSTM, CNN-GRU, XGBoost, and TCN. These models were considered due to their capabilities to cope with time-series data and their proven effectiveness in prior works discussed in [9].

The 1D-CNN is adept at capturing local temporal patterns in sequential data through convolutional operations. This model is considered to serve as a baseline due to its simplicity in processing time-series data. LSTM is a type of RNN (Recurrent Neural Network) designed to learn long-term dependencies in time-series data. It is included here as another baseline model because of its widespread use and success in various time-series prediction tasks. XGBoost (Extreme Gradient Boosting) is another baseline model considered here that uses gradient boosted decision trees to conduct prediction. CNN-GRU is a combined model that integrates the 1D-CNN with the GRU. It utilizes the strength of the CNN to capture spatial patterns and the strength of the GRU to capture temporal dependencies. This combined network is also included as another baseline model due to its high accuracy, as reported in [23]. TCN is another form of convolutional network tailored for time-series data. It was used in the previous study by our research team in [8], and thus it is included here as yet another baseline model.

### 3.2. Window Size

The size of the window used for the input data to the models plays a crucial role in the prediction. If the window size is too short, it fails to provide sufficient input information for constructing a stable prediction model. Conversely, if the window size is excessively long, it leads to little attention to local changes and higher noise, thus diminishing prediction accuracy.

**Figure 3:**
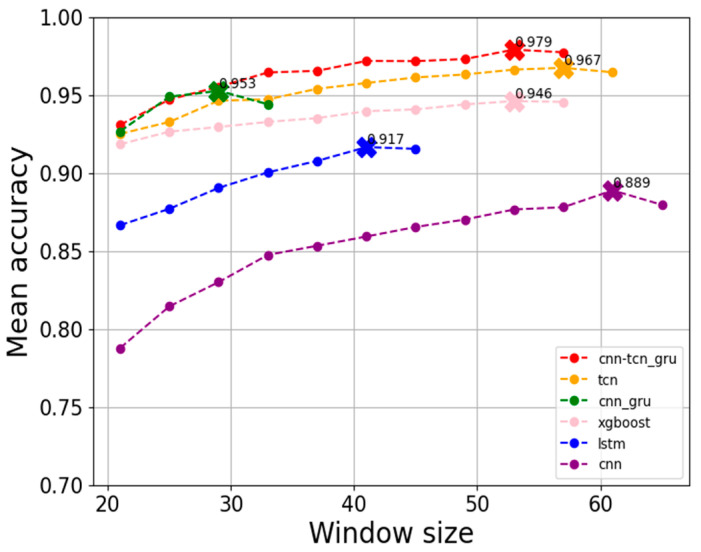
Window size selection.

We started with the window size of 21 units (each unit represents 10 min) as reported in [10] and increased it in steps of 4 units instead of 1 unit in order to save training time by not doing retraining for every single step size. The average prediction accuracy across the prediction horizons from 1 h to 48 h ahead was then considered to set the best window size. The window size was increased till the average prediction accuracy started falling, which was an indication of overfitting. Figure 3 shows the result of the window size selection experiment. As can be seen from this figure and Table 4, the best window size depends on the model used. For the next set of experiments, the best window size of each model was considered for testing. In Figure 3, points marked by ‘**x**’ represent the highest point before the accuracy drops as the window size grows, and the value shown are the accuracies at that point.

### 3.3. Prediction Accuracy

The commonly used metrics of accuracy and F1-score were computed to evaluate the performance of the developed wind turbine icing prediction model. Accuracy measures the portion of all the samples for which the model predicts correctly as indicated by

(20) Accuracy=(TP+TN)/(TP+TN+FP+FN)

where icing state was taken to be positive and normal state to be negative; True Positive (TP) denotes the number of samples that were predicted to be positive, which were actually positive, while True Negative (TN) denotes the number of samples predicted to be negative, which were actually negative. Conversely, False Positive (FP) represents the number of samples predicted to be positive but were actually negative, and False Negative (FN) represents the number of samples predicted to be negative but were actually positive.

F1-score is another measure, which corresponds to the harmonic mean of Precision and Recall as defined in the following equations:

(21) Precision=TP/(TP+FP)

(22) Recall=TP/(TP+FN)

(23) F1−score=2×Precision×Recall/(Precision+Recall)

where Precision denotes the ratio of the samples that are in an actual icing state to those predicted to be in an icing state, and Recall denotes the ratio of the icing state samples that are correctly predicted to all the icing state samples. F1-score is a measure, which is inversely related to the number of incorrectly predicted samples. In other words, the higher the F1-score is, the lower the false prediction errors are.

### 3.4. Computational Aspects

This subsection includes the computational aspects of the model developed in comparison with the existing models. These aspects are shown in Table 5 in terms of the number of parameters, training time, and prediction time for each model on the T4 GPU of Google Colab [24]. The experiments were conducted using Python libraries TensorFlow (version 2.17), NumPy (version 1.26), and scikit-learn (version 1.6) on Google Colab.

Although our model takes more time to train than the other models, it should be noted that the training is carried out offline; that is, it does not play a role in the actual real-time deployment of our prediction solution. The offline training is carried out only once. What matters is how long it takes to perform a prediction as a new data sample becomes available. As shown in Table 5, all the models carry out a prediction within a few milliseconds, which is far shorter than the data or decision update rate of 10 min. In other words, the prediction process, regardless of which model is deployed, operates in real time, noting that real time here means a prediction is made as soon as a new SCADA data sample becomes available.

## 4. Experimental Results and Discussion

### 4.1. Experiment Results

**Figure 4:**
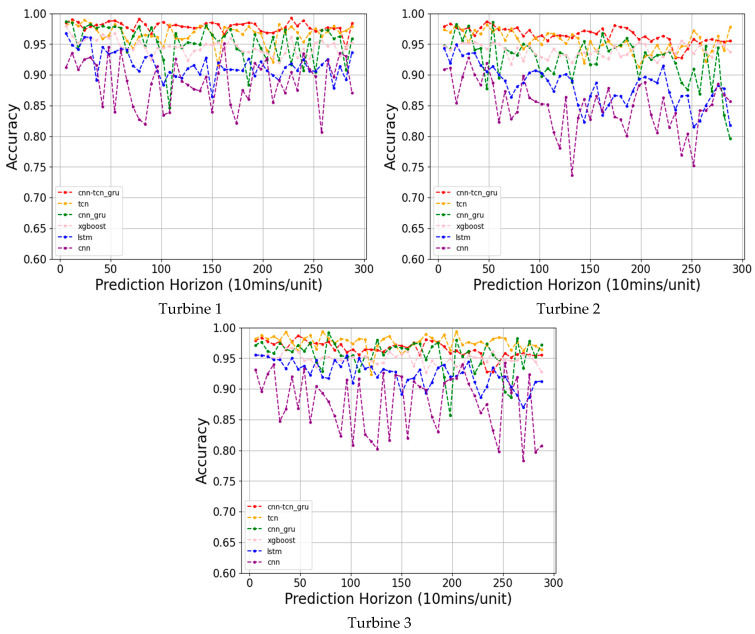
Testing data prediction accuracies from 1 h to 48 h ahead.

Figure 4 as well as Table 6 presents the prediction accuracies and F1-scores of the testing data (time sequence segments of the window size) corresponding to the PCTG and the five baseline models for three wind turbines in the wind farm studied in [11].

**Figure 5:**
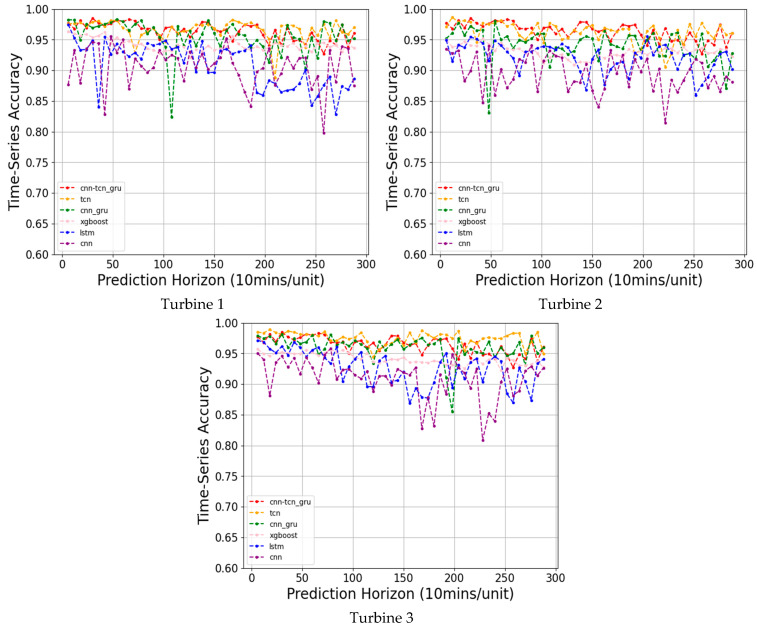
Time-series prediction accuracies from 1 h to 48 h ahead.

Figure 5 as well as Table 7 presents the prediction accuracies and F1-scores across the entire time-series data corresponding to the PCTG and the five baseline models for the same three wind turbines.

The comparison indicates that the developed ensemble PCTG network performs the best for the testing data.

### 4.2. Discussion of Results

The experimental results presented in Table 6 and Table 7 show the superior performance of the PCTG network model in predicting icing compared to the other models: the 1D-CNN, LSTM, XGBoost, CNN-GRU, and TCN. The tables include three different turbines or SCADA datasets. For Turbine 1, the PCTG-net achieved the highest accuracy of 0.9791 and F1-score of 0.9792 with the smallest standard deviations of 0.0082 and 0.0083, respectively. These outcomes indicate that not only is the PCTG more accurate but it is also more consistent across different prediction horizons, outperforming the next-best model (TCN), which provided an accuracy of 0.9674 and an F1-score of 0.9675. For Turbine 2, the PCTG maintained its superior performance with an accuracy of 0.9645 and an F1-score of 0.9649. For Turbine 3, again, the PCTG generated the best accuracy of 0.9810 and an F1-score of 0.9811.

Table 7 extends the analysis where the data were processed in a time-series manner, simulating the way prediction is performed in a real-time or live manner. The results reported in this table confirm the superiority of the PCTG over the other models. Similar to the testing data case, for all the three turbines, the PCTG achieved the highest performance compared with the other models.

Another experiment was conducted for a limited examination of the model performance due to seasonal variations, noting that a comprehensive examination of seasonal variations requires the availability of a multi-year, multi-seasonal dataset, which is not currently available in the public domain. The dataset used here covers the period from January 2023 to June 2023. In this experiment, the testing was repeated for the springtime duration, May to June, instead of the wintertime duration of January to April. As expected, the prediction for the springtime duration produced no icing state or 100% prediction accuracy.

**Figure 6:**
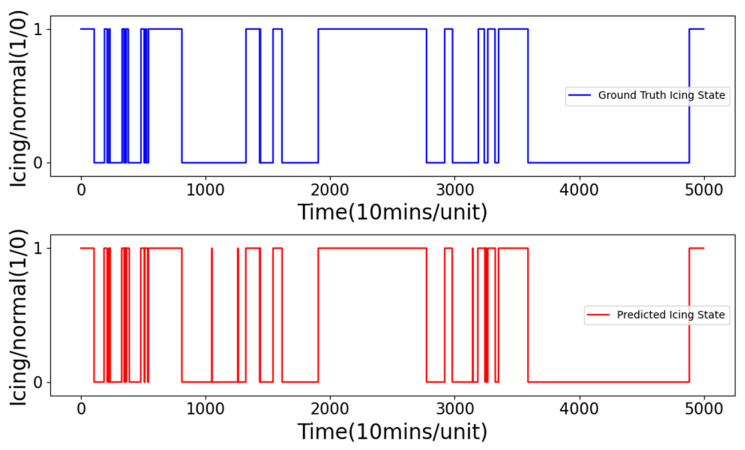
A time-series example showing ground truth and predicted icing states (0: normal state, 1: icing state).

Figure 6 shows an example of the icing state as a time series for a 6 h-ahead prediction. As can be seen from this figure, the predicted icing state is nearly identical to the ground truth icing labels, illustrating the effectiveness of the PCTG when conducting wind turbine icing prediction in a real-time or live manner.

As illustrated in Figure 4 and Figure 5, from 1 h ahead to 48 h ahead for all three turbines, the PCTG outperformed the other models. The superior performance of the PCTG is attributed to its ability to capture both short-term and long-term temporal patterns as well as dependencies on SCADA data, making it a highly effective model for long-term ice prediction or forecasting on wind turbines.

To further show the effectiveness of the PCTG for long-term ice prediction, another experiment was carried out to examine how far ahead the prediction can be made. Such an analysis on ice prediction has not been provided in any previous papers. A model that can predict the farthest time into the future is clearly a more effective model. In this experiment, the breakdown in prediction was defined to be when the average prediction accuracy in the last 5 h became lower than 95%. Table 8 shows the outcome of this experiment. The PCTG network model provided the farthest prediction horizon of 22 days ahead, far more than the other models.

### 4.3. Additional Experiments on Public Domain Dataset

To demonstrate the generalization capability of the PCTG model across different datasets, the public domain dataset from the 2017 China’s Industrial Big Data Competition [25] was also considered. This dataset covers the period from November 2015 to January 2016, focusing on a winter season when icing conditions are prevalent. It consists of the SCADA data samples for 60 days, with each sample labeled as normal or ice. The ratio of normal to ice data samples is approximately 16.5:1, and the data have a sampling time interval of either 7 or 10 s. For consistency, the dataset was resampled to a one-minute interval, and the same feature selection, window size, and training–testing data separation steps carried out for the first dataset were applied to this dataset. As shown in Table 9, the PCTG achieved high accuracies even at 10 days ahead for this dataset as well.

Furthermore, to evaluate the superiority of the PCTG for the long-term prediction of icing, its performance for time-series data was compared with the other models. As shown in Table 10, the PCTG achieved the highest accuracy of 0.99 and the highest F1-score of 0.92.

## 5. Conclusions and Future Enhancements

In this paper, a new combined or ensemble deep learning network model named PCTG is introduced for the purpose of high-accuracy and long-term prediction of icing on wind turbine blades based on SCADA data. This model integrates the advantages of the CNN, TCN, and GRU by incorporating both short-term and long-term temporal patterns as well as dependencies on SCADA variables. The experimentations carried out have shown that this new model achieves high prediction accuracies across long prediction horizons for the two datasets examined. In summary, the newly developed data-driven icing prediction model of PCTG provides higher prediction accuracies and, at the same time, longer prediction horizons than the previously used models for the prediction of icing on wind turbines.

While this study has demonstrated the effectiveness of the developed ensemble model for wind turbine icing prediction, a number of enhancements can be performed in future research. One enhancement involves improving the model’s generalization capability by applying it to a broader range of datasets from various geographical locations. This would allow its adaptability to different climate conditions and turbine structures. Another enhancement involves training the model across multiple years upon the availability of such datasets in order to improve its generalization capability for seasonal variations. The model can also be extended as part of a fusion framework to encompass an entire wind farm consisting of many individual wind turbines by aggregating predictions from multiple turbines to predict the power production capacity of a wind farm due to icing.

## Figures and Tables

**Table 1:** Selected features from SCADA data together with weather, temperature, and relative humidity.

Features
Average power
Gear bearing temperature
Generator speed
Relative humidity
Wind speed
Generator temperature
Nacelle temperature
Blade pitch angle
Weather temperature
Wind direction
Nacelle direction
Ambient temperature

**Table 2:** Ice labelling rules.

Labeling Rules
Temperature < 0 °C
Relative Humidity > 85%
Actual Power < 85% × Power Curve

**Table 3:** Numbers of SCADA data samples.

	Normal	Icing
Training	4588	4588
Testing	1148	1148

**Table 4:** Best window size reached based on average prediction accuracy across prediction horizons.

Network	Best Window Size
1D-CNN	61
LSTM	41
XGBoost	53
CNN-GRU	29
TCN	57
PCTG	53

**Table 5:** Computational aspects.

Model	Parameters	Training Time	Prediction Time
1D-CNN	4705	1 min 39 s	5 ms
LSTM	79,529	2 min 45 s	6 ms
XGBoost	1157	29 s	1 ms
CNN-GRU	55,681	30 min 10 s	12 ms
TCN	6593	1 min 14 s	5 ms
PCTG	40,461	6 min 15 s	7 ms

**Table 6:** Average prediction accuracies and F1-scores of testing data.

		Accuracy		F1-Score	
Turbine 1 testing data	Model	mean	std	mean	std
	1D-CNN	0.8889	0.0376	0.8853	0.0451
	LSTM	0.9166	0.0215	0.9174	0.0222
	XGBoost	0.9461	0.0123	0.9473	0.0123
	CNN-GRU	0.9527	0.0284	0.9522	0.0293
	TCN	0.9674	0.0149	0.9675	0.0149
	PCTG	0.9791	0.0082	0.9792	0.0083
Turbine 2 testing data	Model	mean	std	mean	std
	1D-CNN	0.8494	0.0411	0.8391	0.0549
	LSTM	0.8827	0.0323	0.8748	0.0373
	XGBoost	0.9391	0.0100	0.9407	0.0080
	CNN-GRU	0.9249	0.0362	0.9223	0.0418
	TCN	0.9539	0.0169	0.9531	0.0177
	PCTG	0.9653	0.0124	0.9657	0.0124
Turbine 3 testing data	Model	mean	std	mean	std
	1D-CNN	0.8777	0.0468	0.8704	0.0581
	LSTM	0.9248	0.0204	0.9245	0.0207
	XGBoost	0.9488	0.0048	0.9498	0.0047
	CNN-GRU	0.9554	0.0256	0.9550	0.0270
	TCN	0.9762	0.0117	0.9761	0.0122
	PCTG	0.9821	0.0078	0.9821	0.0079

**Table 7:** Average prediction accuracies and F1-scores of time-series data.

		Accuracy		F1-Score	
Turbine 1 time-series data	Model	mean	std	mean	std
	1D-CNN	0.9065	0.0324	0.7946	0.0557
	LSTM	0.9093	0.0361	0.8091	0.0578
	XGboost	0.9416	0.0096	0.8700	0.0196
	CNN-GRU	0.9560	0.0272	0.8988	0.0534
	TCN	0.9663	0.0160	0.9209	0.0324
	PCTG	0.9777	0.0096	0.9466	0.0211
Turbine 2 time-series data	Model	mean	std	mean	std
	1D-CNN	0.8952	0.0289	0.6587	0.0622
	LSTM	0.9219	0.0229	0.7229	0.0668
	XGBoost	0.9304	0.0197	0.9407	0.0100
	CNN-GRU	0.9411	0.0263	0.7924	0.0723
	TCN	0.9626	0.0163	0.8605	0.0501
	PCTG	0.9648	0.0138	0.8700	0.0450
Turbine 3 time-series data	Model	mean	std	mean	std
	1D-CNN	0.9096	0.0328	0.7292	0.0673
	LSTM	0.9270	0.0289	0.7850	0.0656
	XGBoost	0.9413	0.0118	0.8232	0.0290
	CNN-GRU	0.9600	0.0207	0.8721	0.0536
	TCN	0.9752	0.0105	0.9169	0.0322
	PCTG	0.9784	0.0092	0.9276	0.0274

**Table 8:** Farthest prediction horizon.

Model	Long-Term Prediction Accuracy
1D-CNN	<5 h
LSTM	6 h
XGBoost	14 h
CNN-GRU	28 h
TCN	63 h
PCTG	549 h

**Table 9:** Accuracies of the public SCADA dataset over 10 days.

Prediction Horizons (day)	1	2	3	4	5
Accuracy (%)	99.32	99.48	99.05	99.68	99.74
Prediction Horizons (day)	6	7	8	9	10
Accuracy (%)	99.38	99.42	99.69	99.54	99.47

**Table 10:** Average accuracies and F1-scores of different models for the time-series of the public SCADA dataset at 10 days ahead.

	Model	Mean Accuracy	Mean F1-Score
Public time-series SCADA data	1D-CNN	0.9653	0.8049
	LSTM	0. 9788	0. 8725
	XGBoost	0.9607	0.6893
	CNN-GRU	0.9691	0.8203
	TCN	0.9798	0.8739
	PCTG	0.9886	0.9246

## Data Availability

Data used in this study were provided by the Center for Wind Energy at the University of Texas at Dallas. Additional data were acquired from the publicly available data from the 2017 China’s Industrial Big Data Innovation Competition, at https://www.industrial-bigdata.com/Home (accessed on 15 June 2017).
